# Oral health behavior and oral health service utilization among cancer patients in China: A multicenter cross-sectional study

**DOI:** 10.3389/fonc.2023.1027835

**Published:** 2023-04-05

**Authors:** Ran An, Zitong Wu, Meizi Liu, Yaqin Zhao, Wenfeng Chen

**Affiliations:** ^1^ Teaching and Research Section of Clinical Nursing, Xiangya Hospital Central South University, Changsha, Hunan, China; ^2^ Xiangya School of Nursing, Central South University, Changsha, Hunan, China

**Keywords:** oral health behaviors, oral health service utilization, oral health, cancer patient, cross-sectional study

## Abstract

**Purpose:**

Oral health plays an important role in overall health. But there is scarce information available on oral health behavior and oral health service utilization among cancer patients. This study aimed to evaluate oral health behavior and oral health service utilization among different population groups of cancer patients in China.

**Methods:**

A multicenter cross-sectional study in three tertiary hospitals was conducted to explore the oral health behaviors and oral health service utilization of 162 cancer patients in China.

**Results:**

We investigated a total of 162 cancer patients, 81 from urban and rural areas, respectively. The participant’s ages ranged from 18 and 82 years, mean age was 44.62 years (SD = 15.72). Overall, cancer patients have poor oral health behaviors and limited oral health service utilization. There were statistically significant differences (*p* < 0.05) between urban and rural cancer patients in terms of oral health behaviors, including brushing methods, the use of fluoride toothpaste, the use of dental floss, dental caries, and bleeding gums while brushing teeth. As for oral health service utilization, there were significant differences (*p* < 0.05) between urban and rural cancer patients on regular dental cleaning, the reasons for visiting a dental clinic, and whether they took the initiative to learn about oral health.

**Conclusion:**

The study findings suggest that cancer patients had poor oral health behaviors and limited oral health service utilization, and rural patients perform poorer than their urban counterparts. Oral health education should be provided to cancer patients to improve their oral health behaviors and oral health service utilization.

## 1 Background

Cancer is one of the major challenges facing public health and healthcare systems globally, which poses a heavy burden (1). In 2018, a total number of 18 million new cases have been diagnosed and 8.97 million deaths were attributed to cancer worldwide (1), and cancer is the leading cause of death in China (2).

As great progress has been made in the diagnosis and treatment of cancer, the cancer survival rate has increased dramatically (3), and improving the quality of life remains a unique challenge for these cancer survivors (4). Besides, the decrease in systemic immunity due to disease and treatment modalities such as surgery, radiotherapy, and chemotherapy can have profound effects on the whole and oral health (5), patients are often susceptible to periodontal diseases including periodontitis, loose teeth, tooth decay, mucositis, loss of taste, and bad breath (6). Studies have shown that the incidence of oral mucositis in cancer patients during treatment ranges from 20% to 90% (7, 8), and 37% of head and neck cancer patients experienced dental caries following radiotherapy (9). According to previous studies in other countries, the oral problems of cancer patients are not optimistic, the prevalence of oral health problems including bleeding gums, toothache, mouth ulcers experienced by cancer patients was up to 86.1% (10). More than 36% of survivors had periodontal disease and 15.9% needed dentures, which was higher than the general public, according to data from cancer survivors from Korea (11).

In addition, some studies have shown that poor oral health affects survival in head and neck cancer (12), and poor oral health may impose a financial burden on patients (10). The maintenance of good oral health care is essential for nutrition, recovery, and well-being in cancer patients (13). The impact of oral health on the quality of life of cancer patients has received increasing attention in recent years (14), and it has been demonstrated in numerous studies that oral health is influenced by oral health behaviors and the use of oral health care services (11, 15, 16). To achieve and maintain good oral hygiene, tooth brushing, flossing, and the use of fluoride toothpaste are recommended (17).

However, existing studies show that the oral health behavior of cancer patients is not optimistic (10, 11). A Korean nationwide survey showed that the percentages of cancer patients reporting flossing, mouthwash, interdental brushes, and electric toothbrushes were 17%, 20%, 15.9%, and 5.5%, respectively, and 30.8% of patients reported having an oral examination (11). The overall percentages of Chinese adults who exhibited dental flossing, mouth rinsing, and scaling were 2.6%, 10.3%, and 2.6%, respectively, and only 6.4% of them sought a dentist in the case of gingival bleeding (18). Furthermore, according to the 4th National Oral Health Survey of China (19). The prevalence of the utilization of oral health services in the past 12 months in the subject groups - 3 to 5 years, 12 to 15 years, and 35 to 74 years was 14.6%, 23.6%, and 20.1%, respectively, which is lower than the percent of 26.4% of adult Nigerians (20), and much lower than the percent of 56.2%~65.1% of American adults (21).

At present, studies on oral health mainly focus on children and the elderly in China (22, 23), while cancer patients, as a population with a high prevalence of oral diseases, deserve more attention. This study aims to investigate the oral health behaviors and oral health service utilization of cancer patients.

## 2 Methods

### 2.1 Study design

A multicentral cross-sectional study design was conducted from March 2022 to July 2022 with 162 patients aged from 18 to 82 years old that attended Xiangya Hospital of Central South University, Xiangya Second Hospital, and Xiangya Third Hospital of Central South University, Hunan, China. The three tertiary hospitals in Hunan province with an oncology department, provide comprehensive cancer treatment to thousands of patients each year. Written informed consent was obtained from the participants. The inclusion criteria, specifically, included both male and female adult cancer survivors, aged ≥18 years, who had no evident cognitive impairment and agreed to enroll in the study, and were able to judge independently and fill out the questionnaire. Patients with mental disorders or other serious illnesses and those who were unable to cooperate with the completion of the questionnaire were excluded.

### 2.2 Sampling

The sample size was estimated based on a 95% confidence level and a precision of 5%.

Since this survey is about oral health behaviors and oral health care utilization of cancer patients, and there is no domestic survey for cancer patients, we refer to the national survey data for adults (18). The overall percentages of dental flossing, mouth rinsing, scaling and dental visiting owing to gingival bleeding were 2.6%, 10.3%, 2.6%, and 6.4%, respectively. According to the principle of maximum sample size, the minimum required estimated sample for this study was calculated as 10.3%. Using the formula n=u_2_ap(1-p)/δ (2), the effective response rate is calculated according to 90%, then a total sample of 157 participants was recommended for this study.

### 2.3 Data collection

#### 2.3.1 Socio-demographics questionnaire

A self-reported questionnaire was used to collect information on the patient’s sociodemographic characteristics, such as age, gender, place of residence, marital status, education level, monthly household income, occupational status, smoking status, alcohol consumption status, and monthly household income. We also collected their clinical characteristics information including types of diseases and the length of diagnosis, which were extracted from the electronic medical record system.

#### 2.3.2 Oral health behavior

After reviewing literature and group discussions (24, 25), we developed an 11-item oral health behavior questionnaire, including daily brushing frequency, brushing method, brushing time, frequency of toothbrush replacement, rinsing the mouth after meals, using mouthwash, flossing, using fluoride toothpaste, whether there is caries, whether gums bleed when brushing teeth, and measures to deal with bleeding gums or swollen and painful gums. After a pre-survey of 20 patients, the questionnaire was found to be simple and easy to understand, and it took 5-8 minutes to complete.

#### 2.3.3 Oral health service utilization

The oral health service utilization of participants was self-reported as whether they visited the dentist in the past year (yes or no), whether regular oral examinations are performed (yes or no), whether to have regular dental cleaning (yes or no), the reasons for visiting dentist (toothache is unbearable and medication is not effective, tooth pain is still tolerable, tooth decay is found, regular checkup even without discomfort), and whether they take the initiative to learn about oral health (never, occasionally, often).

### 2.4 Ethical approval and informed consent

All respondents gave informed consent before conducting the interview. All methods were carried out by relevant guidelines and regulations (Declaration of Helsinki). The study protocol was approved by the College of Nursing, Central South University, Nursing and Behavioral Medicine Research Ethics Review Committee (E202295).

### 2.5 Statistical analysis

The data were analyzed using SPSS statistical software version 24.0 (SPSS, Central south university, China). Descriptive data for categorical variables were reported as frequency counts and percentages (%). Differences between groups were calculated using the chi-squared test. All tests of significance were carried out at a *p*-value <0.05. Data were presented in tables and narratives as shown in the result section.

## 3 Results

### 3.1 Subjects

In total, 200 questionnaires were distributed and 168 copies were collected. After checking and excluding 6 cases of invalid questionnaires, 162 valid questionnaires were recovered, with a valid response rate of 81.0%.

The characteristics of the participants from urban and rural areas are shown in **Table 1**, patients living in urban and rural areas are equally divided (81/81). Briefly, the average age of the participants was 44.62 years (SD = 15.72), more than half of them were male (54.9%), and 53.1% of the participants had obtained senior high education; 73.5% were married, and only 27.2% were employed. The study sample predominantly comprised patients suffering from bone tumor (26.5%) and oral cancer (18.5%). In addition, 66% of cancer patients enrolled in the study were newly diagnosed within 1 year. Ethnicity, smoking, alcohol, marital status, sleeping status, cancer type, and time since diagnosis was similar between urban and rural cancer patients. However, there were significant differences (*p* < 0.05) between gender, education level, occupational status, monthly household income, and economic pressure between the two groups. Compared to urban areas, rural areas have more men than women. Generally, patients in rural areas tend to be less educated, unemployed, and have lower monthly incomes, as well as facing more financial struggles.

**Table 1:** Demographic characteristics of cancer patients.

Variables	Urban n (%)	Rural n (%)	Total n (%)	Chi-square	*P* value
**Gender**				15.584	**<0.001**
Male	32 (39.5)	57 (70.4)	89 (54.9)		
Female	49 (60.5)	24 (29.6)	73 (45.1)		
**Ethnicity**				3.287	0.070
Han Chinese	80 (98.8)	74 (91.4)	154 (95.1)		
Ethnic Minority	1 (1.2)	7 (8.6)	8 (4.9)		
**Age group(years)**				2.241	0.326
18~25	17 (21.0)	10 (12.3)	27 (16.7)		
26~55	41 (50.6)	44 (54.3)	85 (52.5)		
≥56	23 (28.4)	27 (33.3)	50 (30.9)		
**Education level**				44.738	**<0.001**
Primary	5 (6.2)	21 (25.9)	26 (16.0)		
Junior Secondary	15 (18.5)	35 (43.2)	50 (30.9)		
Senior Secondary	15 (18.5)	17 (21.0)	32 (19.8)		
College	21 (25.9)	4 (4.9)	25 (15.4)		
Bachelor’s and above	25 (30.9)	4 (4.9)	29 (17.9)		
**Smoking**				2.045	0.153
No	51 (63.0)	42 (51.9)	93 (57.4)		
Yes	30 (37.0)	39 (48.1)	69 (42.6)		
**Alcohol**				0.107	0.743
No	51 (63.0)	53 (65.4)	104 (64.2)		
Yes	30 (37.0)	28 (34.6)	58 (35.8)		
**Occupational status**				27.338	**<0.001**
Full-time	32 (39.5)	12 (14.8)	44 (27.2)		
Unemployed	8 (9.9)	29 (35.8)	37 (22.8)		
Retired	15 (18.5)	5 (6.2)	20 (12.3)		
Other	26 (32.1)	35 (43.2)	61 (37.7)		
**Marital Status**				1.754	0.416
Married	56 (69.1)	63 (77.8)	119 (73.5)		
Unmarried	21 (25.9)	16 (19.8)	37 (22.8)		
Divorced or widowed	4 (4.9)	2 (2.5)	6 (3.7)		
**Sleeping status**				3.002	0.223
Good	36 (44.4)	27 (33.3)	63 (38.9)		
Fair	35 (43.2)	46 (56.8)	81 (50.0)		
Poor	10 (12.3)	8 (9.9)	18 (11.1)		
**Monthly household income (RMB)**				34.399	**<0.001**
≤2000	11 (13.6)	41 (50.6)	52 (32.1)		
2000~5000	21 (25.9)	23 (28.4)	44 (27.4)		
5000~10000	35 (43.2)	15 (18.5)	50 (30.9)		
≥10,000	14 (17.3)	2 (2.5)	16 (9.9)		
**Economic pressure**				39.534	**<0.001**
None	18 (22.2)	2 (2.5)	20 (12.3)		
Lighter	17 (21.0)	5 (6.2)	22 (13.6)		
Fair	28 (34.6)	21 (25.9)	49 (30.2)		
Heavy	14 (17.3)	30 (37.0)	44 (27.2)		
**Cancer type**				7.065	0.422
Lung cancer	6 (7.4)	12 (14.8)	18 (11.1)		
Breast cancer	3 (3.7)	4 (4.9)	7 (4.3)		
Nasopharyngeal Cancer	8 (9.9)	9 (11.1)	17 (10.5)		
Oral Cancer	10 (12.3)	15 (18.5)	25 (15.4)		
Bone Tumor	21 (25.9)	22 (27.2)	43 (26.5)		
Glioma	12 (14.8)	7 (8.6)	19 (11.7)		
Lymphoma	8 (9.9)	4 (4.9)	12 (7.4)		
Other	13 (16.0)	8 (9.9)	21 (13.0)		
**Time since diagnosis(years)**				0.259	0.879
≤1	53 (65.4)	54 (66.7)	107 (66.0)		
1~5	18 (22.2)	19 (23.5)	37 (22.8)		
≥5	10 (12.3)	8 (9.9)	18 (11.1)		

The significance level was set at 0.05. All statistically significant ones have been marked in bold.

**Table 2** shows the oral health behaviors among urban and rural cancer patients. Statistically significant differences were found between rural and urban cancer patients in brushing methods, dental flossing, fluoride toothpaste, and self-reported dental caries (*p* < 0.05). The majority of respondents (75.3%) brush their teeth twice or more daily, and more than half of patients (59.3%) brush with no fixed method. Up to 74.7% of patients do not floss, with more participants in rural areas than in cities. Over half of the patients (59.3%) are not aware of fluoride toothpaste. About one-third (30.9%) of the participants self-reported having dental caries, 32.1% of the respondents indicated that they were not sure if they had dental caries, and 37% of the patients self-reported having no dental caries.

**Table 2:** Oral health behaviors between urban and rural cancer patients.

Variables	Urban n (%)	Rural n (%)	Total n (%)	Chi-square	P value
**Daily brushing frequency**				2.124	0.547
0	1 (1.2)	4 (4.9)	5 (3.1)		
1 time	17 (21.0)	18 (22.2)	35 (21.6)		
2 times	54 (66.7)	49 (60.5)	103 (63.6)		
More	9 (11.1)	10 (12.3)	19 (11.7)		
**Brushing method**				12.722	**0.005**
Brush horizontally	9 (11.1)	27 (33.3)	36 (22.2)		
Brush vertically	20 (24.7)	11 (13.6)	31 (19.1)		
Brush in a circle	11 (13.6)	11 (13.6)	22 (13.6)		
No fixed method	41 (50.6)	32 (39.5)	73 (45.1)		
**Brushing time**				1.222	0.543
1 minute or less	10 (12.3)	15 (18.5)	25 (15.4)		
2 minutes	41 (50.6)	37 (45.7)	78 (48.1)		
≥ 2 minutes	30 (37.0)	29 (35.8)	59 (36.4)		
**Frequency of toothbrush replacement**				2.109	0.550
Never	9 (11.1)	12 (14.8)	21 (13.0)		
Semi-annually	14 (17.3)	16 (19.8)	30 (18.5)		
2~3 months	46 (56.8)	37 (45.7)	83 (51.2)		
1 month	12 (14.8)	16 (19.8)	28 (17.3)		
**Rinsing the mouth after meals**				6.004	0.111
Never	33 (40.7)	46 (56.8)	79 (48.8)		
1 time per day	16 (19.8)	13 (16.0)	29 (17.9)		
2 times a day	16 (19.8)	7 (8.6)	23 (14.2)		
≥3 times a day	16 (19.8)	15 (18.5)	31 (19.1)		
**Use mouthwash**				0.121	0.727
No	57 (70.4)	59 (72.8)	116 (71.6)		
Yes	24 (29.6)	22 (27.2)	46 (28.4)		
**Flossing**				7.347	**0.007**
No	53 (65.4)	68 (84.0)	121 (74.7)		
Yes	28 (34.6)	13 (16.0)	41 (25.3)		
**Use fluoride toothpaste**				12.254	**0.002**
Yes	22 (27.2)	22 (27.2)	44 (27.2)		
No	11 (13.6)	29 (35.8)	40 (24.7)		
Don’t know	48 (59.3)	30 (37.0)	78 (48.1)		
**Whether there is caries**				7.356	**0.025**
Yes	27 (33.3)	23 (28.4)	50 (30.9)		
No	22 (27.2)	38 (46.9)	60 (37.0)		
I don’t know	32 (39.5)	20 (24.7)	52 (32.1)		
**Gums bleed when brushing teeth**				1.229	0.268
No	39 (48.1)	32 (39.5)	71 (43.8)		
Yes	42 (51.9)	49 (60.5)	91 (56.2)		
**When brushing bleeding or gums are swollen and painful**				5.729	0.126
No treatment needed	43 (53.1)	55 (67.9)	98 (60.5)		
Go back to the dentist when you have time	17 (21.0)	14 (17.3)	31 (19.1)		
Seek immediate medical care	10 (12.3)	3 (3.7)	13 (8.0)		
Take medication or other	11 (13.6)	9 (11.1)	20 (12.3)		

The significance level was set at 0.05. All statistically significant ones have been marked in bold.

**Table 3** below shows the oral health service utilization between urban and rural cancer patients. There were statistically significant differences (*p* < 0.05) between urban and rural cancer patients in reasons for regular dental cleaning, dental visiting, and taking the initiative to learn about oral health.

**Table 3:** Oral health service utilization between urban and rural cancer patients.

Variables	Urban n (%)	Rural n (%)	Total n (%)	Chi-square	P value
**Visited the dentist in the past 12 months**				0.359	0.549
No	64 (79.0)	67 (82.7)	131 (80.9)		
Yes	17 (21.0)	14 (17.3)	31 (19.1)		
**Regular oral examination**				1.786	0.181
No	60 (74.1)	67 (82.7)	127 (78.4)		
Yes	21 (25.9)	14 (17.3)	35 (21.6)		
**Regular dental cleaning**				4.410	**0.036**
No	58 (71.6)	69 (85.2)	127 (78.4)		
Yes	23 (28.4)	12 (14.8)	35 (21.6)		
**Reasons for visiting the dentist**				10.206	**0.037**
Never	14 (17.3)	26 (32.1)	40 (24.7)		
Toothache is unbearable and medication is not effective	28 (34.6)	27 (33.3)	55 (34.0)		
Tooth pain is still tolerable	26 (32.1)	22 (27.2)	48 (29.6)		
Tooth decay is found	5 (6.2)	5 (6.2)	10 (6.2)		
Regular checkups even without discomfort	8 (9.9)	1 (1.2)	9 (5.6)		
**Take the initiative to learn about oral health**				12.702	**0.002**
Never	24 (29.6)	45 (55.6)	69 (42.6)		
Occasionally	49 (60.5)	34 (42.0)	83 (51.2)		
Often	8 (9.9)	2 (2.5)	10 (6.2)		

The significance level was set at 0.05. All statistically significant ones have been marked in bold.

A majority of patients (80.9%) didn’t visit the dentist in the past year, patients who didn’t have a regular oral examination and dental cleanings each accounted for 78.4%, and most of the reasons for visiting the dentist were toothache (66.7%), only 20% of patients reported regular dental cleanings, the percentage of a dental cleaning in urban areas and rural areas are 28.4% and 14.8%, respectively. More than half (60.5%) of patients self-reported that they occasionally took the initiative to learn about oral health.

**Table 4** shows the results of binary and multivariate logistic regression analyses of outcomes variables (i.e., oral health behaviors and oral health service utilization) across urban and rural cancer patients controlling for demographics. For the binary outcome variables, a binary logistic regression analysis was performed; otherwise, a multivariate logistic regression analysis was performed. Regarding the method of brushing teeth, there is an urban-rural disparity (*p*=0.01) with 50.6% of urban patients reporting no fixed method of brushing teeth compared to 39.5% for rural patients. As for flossing, there is an urban-rural disparity (*p*=0.08) with 34.6% of urban patients reporting flossing compared to 16.0% for rural patients. When it comes to fluoride toothpaste, there is an urban-rural disparity (*p*=0.042) with 39.5% of urban patients reporting not sure whether it is fluoride toothpaste compared to 24.7% for rural patients. An urban-rural disparity also emerged for dental caries. More rural patients than urban patients reported no dental caries. Concerning dental cleaning, 28.4% of urban patients reported regular dental scaling compared to 14.8% of rural patients.

**Table 4:** Binary and multivariate logistic regression analyses of oral health behaviors and oral health service utilization across urban and rural cancer patients controlling for demographics.

Independent variable		β	SE	β’	*P*	*EXP(B)*	OR value	
							Upper limit	Lower limit
Brushing method	Brush vertically	1.312	0.708	3.433	0.064	3.715	0.927	14.890
	Brush in a circle	1.479	0.766	3.728	0.054	4.391	0.978	19.713
	No fixed method	1.534	0.598	6.572	**0.010**	4.634	1.435	14.968
Flossing		1.016	0.382	7.067	**0.008**	2.763	1.306	5.847
Fluoride toothpaste	No	-0.278	0.604	0.211	0.646	0.758	0.232	2.475
	I don’t know	1.050	0.517	4.126	**0.042**	2.858	1.038	7.874
Dental caries	No	-1.068	0.529	4.073	**0.044**	0.344	0.122	0.970
	I don’t know	0.353	0.531	0.442	0.506	1.423	0.503	4.029
Dental Cleaning		0.824	0.398	4.285	**0.038**	2.280	1.045	4.976
Dental visiting	Toothache is unbearable and medication is not effective	-0.510	0.575	0.785	0.376	0.601	0.195	1.855
	Tooth pain is still tolerable	-0.685	0.603	1.293	0.255	0.504	0.155	1.642
	Tooth decay is found	0.838	0.867	0.934	0.334	2.311	0.422	12.643
	Regular checkups even without discomfort	2.108	1.237	2.904	0.088	8.232	0.729	92.971
Take the initiative to learn about oral health	Occasionally	-0.095	0.442	0.046	0.830	0.909	0.383	2.161
	Often	0.909	0.989	0.846	0.358	2.483	0.357	17.248

The significance level was set at 0.05. All statistically significant ones have been marked in bold.

## 4 Discussion

This study examined oral health behavior and oral health utilization among cancer patients in China. Despite nationally stated goals to reduce oral health disparities, our study confirms a persistent gap between urban and rural cancer patients. We found that rural cancer patients were more likely to deliver poor oral health behaviors and have limited oral health utilization compared to rural cancer patients. These findings suggest the need for better strategies to improve oral health for both urban and rural cancer patients.

Numerous studies have shown that proper brushing, appropriate use of dental care products, and regular dental checkups are the main ways to maintain oral health (24, 26). The results of this study showed that 74.6% of cancer patients brushed their teeth twice a day or more, which is much than the ratio of 36.1% of middle-aged people revealed in the fourth national survey of China (16), and higher than the percent of 66.2% of Peruvian adults (24), but less than the percent of 83.7% of a survey in Korea cancer patients (11). Although most oncology patients can brush their teeth twice a day or more, it is worth noting that there were large differences in brushing methods, with 24.6% choosing horizontal brushing, 16.4% choosing vertical brushing, only 13.4% choosing rotary brushing, and nearly half (45.5%) not having a fixed brushing method. Correct and proper brushing method helps to control plaque to maintain good oral hygiene, thus largely preventing or controlling caries and periodontal disease (27). The modified Bass brushing method or the vertical brush rotation method is recommended by dental specialists (28, 29). In addition, those who could brush for two minutes or more each time accounted for 84.4%. Most patients (51.2%) change their toothbrushes every two to three months, 18.5% of patients changed their toothbrushes every six months, while there were 13% of the patients never changed their toothbrushes, the results were similar to a previous survey of Pakistani medical students (30).

The “Chinese Residents’ Guide to Oral Health Behavior” manual for health care professionals recommends the use of comprehensive oral cleaning behaviors including brushing, rinsing, and flossing to maximize plaque removal and control plaque growth [15]. The awareness and utilization of oral health care products, except for the most basic toothbrush and toothpaste, the behaviors of rinsing the mouth, using mouthwash, flossing, and using fluoride toothpaste are limited. Nearly half of the patients (48.8%) never rinse their mouth after meals, and only 28.4% of patients choose to rinse with mouthwash, which was much lower than the ratio of 47% of the Swedish adult population (31). Studies have shown that a mouth rinse administered intraorally causes salivary and mouth rinse properties to change, and the changes in those factors can affect preventive and therapeutic effects, as well as oral health (32). Researchers have shown that dentist recommendations play an important role in mouthwash use, so dental professionals should play a greater role in advising patients who need to use mouthwash to pick the most appropriate product (31).

Flossing is the use of threads made from synthetic fibers to clean plaque and food debris from the adjacent surfaces of teeth and interdental spaces, but our study showed that only 25.3% of patients flossed, and there was an urban-rural difference, with a much lower percentage of patients from rural areas flossing than those from urban areas. Previous studies have shown that flossing is influenced by flossing self-efficacy (33, 34), so patients should be encouraged to floss and strategies to overcome barriers should be discussed by dental professionals.

Although it is recognized that fluoride toothpaste can greatly reduce the incidence of dental caries (35), it is surprising that nearly half of the patients (48.1%) did not know whether the toothpaste used as fluoride toothpaste or not, and unexpectedly, it is also surprising that the urban patients were less aware of fluoride toothpaste than the rural patients. About one-third (30.9%) of the participants self-reported having dental caries, 32.1% of the respondents indicated that they were not sure if they had dental caries, and another 37% of the patients self-reported having no dental caries. However, the Fourth National Oral Health Survey (2015-2016) in mainland China (36) showed that the periodontitis rates of adults were 52.8%~69.3%, and the prevalence of dental caries among Chinese preschool children was as high as 62.5% (37), and a systematic review shows that the caries rate has been gradually increasing in recent years, from 36.4% in the 1980s to 53.1% in 2010s (38). However, despite the high incidence of dental caries and periodontal disease, only a minority of residents seek treatment, which remains a problem that needs to be addressed (39). Since the use of fluoride toothpaste is one of the important ways to prevent caries (35), it is still necessary to increase the awareness and usage of fluoride toothpaste.

More than half of the patients (56.2%) experienced gums bleeding when brushing their teeth. The prevalence of self-reported gingival bleeding is consistent with previous studies, with a survey of adults in Hong Kong (15) showing a prevalence of 62.2% and a survey of adolescents aged 12-15 years in Jiangxi showing an incidence of 66.5% (40). However, most patients (60.5%) believe that bleeding gums or swollen gums do not need to be treated, nearly one-fifth (19.1%) of the patients chose to delay medical treatment, and 12.3% of them chose to take their medication or other, only 8% of patients would seek immediate medical care. Although there is no statistical difference, we can see that the incidence of gingival bleeding during tooth brushing is higher in rural patients than in urban patients. The difference may originate from their lack of knowledge about oral health, so they didn’t realize that gum bleeding may be a symptom of other systemic diseases. Bleeding gums are an early sign of periodontal disease, and its association with periodontal health has been studied extensively in the literature (41). Patients with bleeding gums should receive advice and encouragement from professionals, especially if they have a visible periodontitis infection, poor dental awareness or behaviors, and a poor lifestyle. This will allow them to take action and improve their gum health.

Regarding the utilization of oral health care services, our survey showed that only19.1% of patients visited the dentist in the past 12 months, which is consistent with the percentage of 20.1% of the 4th National Oral Health Survey of China (19), and 80.9% of patients did not visit the dentist in the past 12 months, which is higher than that of 69.2% of cancer patients in Korea (11). Overall, the patients who didn’t have regular oral examinations and dental cleanings each accounted for 78.4%. Although participants from urban areas performed better on dental scaling behavior than those from rural areas, none of them achieved the desired level. This reason may stem from differences in economic and medical resources between rural and urban areas, and the survey in this study also showed that participants in rural areas generally had lower economic income and education, who did not place enough emphasis on oral health had a significant lack of understanding of oral health. A prospective longitudinal study showed that regular dental cleanings prevented tooth loss in older adults (42) and that early and consecutive dental scaling prevents the development of Parkinson’s disease (43). Nevertheless, a study in Brazil (44)showed that the implementation of a national dental scaling policy could indeed increase the rate of scaling among the population but would exacerbate the disparity in socioeconomic inequalities in dental scaling, and therefore universal dental coverage should be considered.

As for the reasons for visiting a dentist, only 5.6% had regular checkups without discomfort, which is lower than the percentage of 19% of a survey of older Chinese and much lower than the percentage of 71.6%~77% of adults in America (21), and there is an urban-rural disparity (45). This may be explained by the fact that dental care is mostly excluded from healthcare coverage in China (45), which means more financial stress for oral health, and compared to oral health, cancer patients are more concerned about the effect of cancer treatment modalities and its effects (46). In addition, perhaps patients have little knowledge of oral health care and a low willingness to take care of oral health, which affects oral health practices (47). Regular dental visits and routine prevention may offer more opportunities for oral health education and reinforcement, which may improve oral health. Since rural cancer patients visit the dentist less, this highlights the importance for dentists to view each visit as a teachable opportunity to promote positive oral health behaviors and improve their knowledge about oral health.

Our survey showed that the majority of patients went to the dentist because of oral discomfort (59.8%), especially swollen and sore gums (59.6%), which is consistent with a survey of adults in Peru (25), but much higher than the ratio of 23%~28.4% of American adults (21). Only 5.6% of patients were able to have regular oral examinations, even without uncomfortable symptoms, which indicated that most of them chose to respond negatively to oral problems and might seek treatment in uncomfortable situations. The survey results suggested that oral health education for cancer patients should focus on guidance on developing good habits of regular dental visiting, and actively seeking medical treatment.

In terms of taking the initiative to learn about oral health-related knowledge, 42.6% of patients never learned, 51.2% learned occasionally, and only 6.2% thought they took the initiative to learn it frequently, indicating that, to some extent, the awareness and behavior of oral health care of cancer patients in China are poor, and do not pay enough attention to oral health. Since it plays an important role in oral health, it is imperative to enhance their knowledge of oral health.

Overall, this study indicates that oral health behaviors among cancer patients, especially those living in rural areas, need to be improved, besides, basic oral hygiene habits, knowledge and use of oral health care products, utilization of oral health care services, and awareness of oral health knowledge still need to be enhanced.

## 5 Limitations

This study is not without limitations, Firstly, due to the cross-sectional nature of the study, cause and consequence cannot be distinguished. Besides, self-rated oral health questionnaires may be affected by recall bias, and the study used a convenience sample of three large general hospitals in Hunan, all of them running under the same management umbrella. The results might have been different if the other organizations had been involved in the study. Furthermore, the study was conducted in the oncology wards without including cancer survivors in the home recovery period, so conclusions should be taken with caution. Moreover, it is worth noting that some oncology patients, especially those with head and neck tumors, rinse their mouths or brush their teeth during radiotherapy under medical prescription, which may be different from their oral health habits previously, or due to the impact of some specific oncology treatment modalities on oral complications such as oral mucositis, oral pain, and dry mouth, among them especially in head and neck radiotherapy patients and other oncology chemotherapy patients, which may affect their daily oral health behaviors. Finally, many factors affect individual health behaviors, and the association and difference between oral health awareness and practical behaviors should be considered, and this study did not investigate the overall health status of patients.

## 6 Conclusion

This study found that cancer patients, both urban and rural patients have poor oral health behaviors and limited oral health service utilization. In some aspects, patients in rural areas performed worse oral health behaviors. Furthermore, many patients never visited the dentist, only a minority of patients have regular dental cleanings or oral exams. Therefore, oral health education should be provided to cancer patients and their caregivers, and oral health assistance and oral assessment should be provided to patients with poor oral health awareness and oral health status to improve their oral health awareness and behavior.

## Data availability statement

The original contributions presented in the study are included in the article/supplementary material. Further inquiries can be directed to the corresponding author.

## Ethics statement

The studies involving human participants were reviewed and approved by the study protocol was approved by the College of Nursing, Central South University, Nursing and Behavioral Medicine Research Ethics Review Committee (E202295). The patients/participants provided their written informed consent to participate in this study.

## Author contributions

RA and WC were responsible for the conception and design of the study. RA, ZW, and ML performed the data collection. RA and ML carried out the analysis of the data and the interpretation of the results. RA drafted the manuscript, with Muhammad Sohaib and WC providing critical revisions. All authors contributed significantly, and read and approved the final manuscript.
